# Knowledge, attitudes, and beliefs about psychotropic medication among patients with mental disorders

**DOI:** 10.1192/j.eurpsy.2024.1718

**Published:** 2024-08-27

**Authors:** M. Abdelkefi, R. Feki, I. Gassara, S. Omri, N. Smaoui, N. Charfi, L. Zouari, J. Ben thabet, M. Maalej bouali, M. Maalej

**Affiliations:** Psychiatry C department, Hedi Chaker university hospital, sfax, Tunisia

## Abstract

**Introduction:**

Non-adherence to treatment is a major concern for mental health professionals. Knowledge of prescribed medications can influence patients’ willingness to adhere to them.

**Objectives:**

The aim of this study was to assess the knowledge of patients with mental disorders about their prescribed medication and to evaluate their attitudes and beliefs toward treatment.

**Methods:**

Our quantitative descriptive study involved 52 patients hospitalized in the psychiatric “C” department of the Hedi Chaker University Hospital in Sax between the 23^rd^ and 30 October 2023. Excluded were aggressive patients, those who were unable to communicate, and those who refused to participate. Thirty-nine patients were included. For each patient, we collected sociodemographic, clinical, and disease progression data, as well as information, beliefs, and attitudes concerning the prescribed treatment.

**Results:**

The mean age of our patients was 35.49 ± 10.24 years, with an exclusively male sample. Most patients had no occupation (69.2%). Only 10.3% were married. Over half of the patients had achieved primary school (61.5%) and lived in rural areas (64.1%). The mean duration of the mental disorder was 10.69 ± 9.07 years. Patients were hospitalized 3.62 times on average. The most frequent diagnoses were schizophrenia (35.9%) and bipolar disorder (33.3%). More than half of the patients (61.5%) knew the color and shape of the prescribed medication, and 48.2% knew the name and dose.

The source of treatment information was mainly doctors (33.3%) and family members (15.4%). Adherence to treatment was poor in 69.2% of cases. The majority of patients denied stopping treatment and 12.8% reported that they stopped treatment because of financial difficulties. Twenty-four patients confirmed that taking the treatment made people see them differently and that they preferred not to reveal they were taking it. Two-thirds of patients reported that the treatment relaxed them (71.8%) but could be stopped when they felt better (69.2%).

**Conclusions:**

It is essential for mental health professionals to develop and implement effective intervention strategies that maximize therapeutic impact and reduce the risk of relapse.

**Disclosure of Interest:**

None Declared

